# Feasibility, user experiences, and preliminary effect of *Conversation Cards for Adolescents©* on collaborative goal-setting and behavior change: protocol for a pilot randomized controlled trial

**DOI:** 10.1186/s40814-019-0533-3

**Published:** 2019-12-18

**Authors:** M. Kebbe, A. Farmer, M. P. Dyson, S. D. Scott, T. L. F. McHugh, S. Lappa, H. Rajani, T. Ladha, B. Islam, L. Jacoby, F. Nasir, K. Talwar, J. L. Wincott, M. Zhang, G. D. C. Ball

**Affiliations:** 1 Department of Pediatrics, Faculty of Medicine & Dentistry, 4-515 Edmonton Clinic Health Academy, University of Alberta, 11405 – 87 Avenue, Edmonton, Alberta T6G 1C9 Canada; 2 Department of Agricultural, Food, and Nutritional Science, Faculty of Agricultural, Life & Environmental Sciences, University of Alberta, Edmonton, Alberta Canada; 3 Faculty of Nursing, University of Alberta, Edmonton, Alberta Canada; 4 Faculty of Kinesiology, Sport, and Recreation, University of Alberta, Edmonton, Alberta Canada; 5 Northeast Community Health Centre, Edmonton, Alberta Canada

**Keywords:** Adolescent, Health services, Life style, Obesity, Pilot projects

## Abstract

**Background:**

Adolescents and providers can benefit from practical tools targeting lifestyle modification for obesity prevention and management. We created *Conversation Cards for Adolescents©* (CCAs), a patient-centered communication and behavior change tool for adolescents and providers to use in clinical practice. The purpose of our study is to (i) assess the feasibility of CCAs in a real-world, practice setting to inform full-scale trial procedures, (ii) assess user experiences of CCAs, and (iii) determine the preliminary effect of CCAs on changing behavioral and affective-cognitive outcomes among adolescents.

**Methods:**

Starting in early 2019, this prospective study is a nested mixed-methods, theory-driven, and pragmatic pilot randomized controlled trial with a goal to enroll 50 adolescents (13–17 years old) and 9 physicians practicing at the Northeast Community Health Centre in Edmonton, Alberta, Canada. Adolescents will collaboratively set one S.M.A.R.T. (specific, measurable, attainable, realistic, timely) goal with their physician to implement over a 3-week period; however, only those randomized to the experimental group will use CCAs to inform their goal. Outcome assessments at baseline and follow-up (3 weeks post-baseline) will include behavioral, affective-cognitive, and process-related outcomes.

**Discussion:**

In examining the feasibility, user experiences, and preliminary effect of CCAs, our study will add contributions to the obesity literature on lifestyle modifications among adolescents in a real-world, practice setting as well as inform the scalability of our approach for a full-scale effectiveness randomized controlled trial on behavior change.

**Trial registration:**

ClinicalTrials.gov Identifier: NCT03821896.

## Introduction

The Task Force appointed by the Endocrine Society to formulate evidence-based clinical practice guidelines for pediatric obesity deemed the prevention and management of obesity in adolescents a high priority [1]. Identified recommendations placed particular attention on the importance of providers in prescribing and supporting a healthy lifestyle through the use of behavior change techniques such as general communication skills and shared-decision making (SDM) [1]. SDM is a patient-centered technique that actively engages patients in the evaluation of available treatment options and in the treatment decision-making process [2]. It is said to be comprised of a pre-decisional model focused on collaborative deliberation, followed by an act of determination for post-decisional outcomes [3]. A desirable strategy for patients and providers to reach agreement on a decision may involve collaborative goal-setting, which is the most widely used technique for behavior change in pediatric obesity interventions [4]. Given the maximized effectiveness of pairing SDM with goal-setting in patients with chronic diseases [5, 6], the inadequacy of education and knowledge alone in changing behavior [7], and adolescents’ increasing developmental capacity to make decisions, these techniques may hold promise in facilitating lifestyle change across a range of behaviors in adolescents through patient-centered goals and actions.

While providers recognize the problem in pediatric obesity and acknowledge their responsibility in addressing weight [8–10], they are often not opportunistic in the prevention and management of obesity through lifestyle interventions. This is likely due to a combination of factors, including high practice workload and a lack of training [11–13] and competency [14], and is explained, at least in part, by empirical data, where only 4.3% of physicians in the USA reported receiving specialty training in obesity [15]. Further, there is little evidence to support the use of goal setting between providers and patients, including adolescents, in early-intervention settings despite recommendations for doing so [16–18]. These data pave the way for practical tools and resources as useful additions to providers’ menu of consultation services. Particularly, rather than direct discussions on weight, lifestyle-based tools that incorporate collaborative goal-setting may maximize providers’ and adolescents’ self-efficacy for lifestyle modification.

Given these points, our team developed a bilingual (English and French) communication and behavior change tool called *Conversation Cards for Adolescents©* (CCAs). We believe that CCAs may have value in supporting providers in delivering health services for adolescents with and without obesity, providing a novel, patient-centered tool to encourage productive conversations about healthy lifestyle behavior changes. Rooted in the social ecological model [19] and the social cognitive theory [7], CCAs capitalize on goal-setting as a mechanism for self-regulation and self-efficacy [7], both of which are often used to close the intention to action gap. A recent systematic review encouraged the conduct of high-quality studies to determine the effectiveness of personalized care planning on goal achievement as set by patients themselves, as opposed to providers or researchers [20]. However, the feasibility of intervention-based programs in promoting healthy lifestyles among adolescents, particularly through the use of tools, message tailoring, and goal-setting, should be explored first. Our aim in conducting this pilot randomized controlled trial (RCT) is to (i) assess the feasibility of CCAs in a clinical setting to inform full-scale trial procedures, (ii) assess user experiences of CCAs, and (iii) determine the preliminary effect of CCAs on changing behavioral and affective-cognitive outcomes among adolescents. Specifically, our primary objective is to assess the feasibility of using CCAs in a real-world, clinical setting to inform full-scale RCT procedures, including time needed to train physicians, acceptability of the proposed design and procedures, acceptability and completeness of recruitment and data collection methods, participation and attrition levels, collaborative goal-setting with adolescents, barriers to maintaining delivery of implementation over the trial period, and sample size estimation. Secondary objectives include assessing user experiences (adolescents and physicians) related to CCAs and determining the preliminary effect of CCAs on changing behavioral and affective-cognitive outcomes among adolescents.

## Methods

### Study design

Our study, which will begin recruitment in spring 2019 (estimated study completion date in April 2020), is a nested mixed-methods, theory-driven, and pragmatic pilot RCT [21, 22] involving adolescents and physicians. The Standard Protocol Items: Recommendations for Interventional Trials (SPIRIT) checklist for this protocol paper is included in Additional file 1. We will follow recommendations for reporting of treatment fidelity in behavioral interventions to manage pediatric obesity [23] and the Consolidated Trials of Reporting Trials (CONSORT) extension for pragmatic trials [24] in reporting our findings.

#### Rationale for a mixed-methods, pragmatic trial

Attempting to mimic real-world settings as closely as possible, pragmatic RCTs are an excellent approach to maximize trial validity and usefulness in health care settings other than the one they are conducted in [25]. Our trial will incorporate the following pragmatic components: use of an existing clinical practice, minimal patient selection criteria, use of real-world adolescent patients, and use of existing practices for recruitment, eligibility assessment, and follow-up procedures [25]. Further, adopting a mixed-methods approach will allow us to assess intervention components and how they interact with one another through triangulation, complementarity, expansion, and development [26].

### Setting

We will establish collaborations with the Northeast Community Health Centre (NECHC, Alberta Health Services, Edmonton, Alberta, Canada). The NECHC offers an academic primary and secondary clinical care setting, here-in referred to as an “early-intervention setting.” Staff at the NECHC include administrative support, nurses, a social worker, and consulting physicians. All participating physicians (SL, HR, TL, BI, LJ, FN, KT, JLW, MZ) are pediatricians who offer general and specialty clinical services for 1–18-year-olds and their families, many of whom are refugees and new Canadians living in urban areas of Edmonton. The pediatric and adolescent clinics contain the main clinic space with a waiting area and eight exam/counseling rooms and offers in-person and telehealth services.

### Inclusion/exclusion criteria

Adolescents will be eligible to participate if they are 13–17 years old, have the developmental and language capacities to complete our intervention (English literacy and comprehension), and are interested in setting a lifestyle/behavioral goal related to improving diet, physical and sedentary activities, sleep, relationships, or mental health. These eligibility requirements will be confirmed by our research team members during recruitment by consulting with administrative/clinical staff (developmental and language capacities) and adolescents (interest in goal-setting). All physicians delivering care to adolescents at the NECHC will be eligible and invited to participate.

### Recruitment

Administrative/clinical staff will approach families and adolescents to gauge interest in our study and obtain verbal consent for contact by the study coordinator (MK). MK will be present on-site to verify eligibility (developmental and language capacities), explain the study to families in detail, and facilitate written assent (adolescents) and consent (parents) procedures. As per existing practice at the NECHC, administrative/clinical staff will measure adolescents’ height and weight on the day of their medical appointment before they see their physician; nurses will liaise with MK to provide demographic and anthropometric data. Physicians will be recruited by email or verbal invitation through existing relationships with research team members. Physicians will be free to accept or decline participation following a brief description of the proposed study.

We will adhere to a number of evidence-based strategies to recruit and retain families [27], including (i) clearly describing study expectations and commitments to families at the time of enrollment, (ii) using families’ preferred mode of contact (e.g., telephone, text message) for correspondence, (iii) ensuring families understand the distinction between research and their clinical care, (iv) confirming families’ understanding of the value of their study participation, (v) establishing an ongoing mutual understanding of study expectations, commitments, and progress to administrative/clinical staff, and (vi) offering gift cards as tokens of appreciation ($25 Visa gift card per adolescent; $25 Amazon gift card per physician and administrative/clinical staff).

### Randomization and allocation procedures

Adolescents will be randomly allocated to one of two groups (experimental or control) with a 1:1 allocation ratio and using randomly varied permuted blocks of 2 and 4. Participant randomization will be performed in REDCap® (Research Electronic Data Capture) using allocation tables that were generated by the Data Coordinating Centre statistician from the Women and Children’s Health Research Institute (University of Alberta). REDCap® maintains an automated audit trail, which includes the assigned study identification number, treatment allocation, and date and time of the transaction. The study coordinator, who will undertake study assessments with adolescents, will have access to REDCap® on-site to randomize and provide allocations to adolescents; allocation concealment will be ensured as the randomization service does not release the code for the randomization and the allocator will have no prior knowledge about the random sequence.

### Trial interventions

#### Physician training

To ensure a comparable level of understanding and communication skills, research team members (MK, GDCB) will hold two in-person orientation sessions (duration 1 h/each) with participating physicians that include discussions and decisions related to the study protocol such as intervention design, process, and logistics. We will also hold one in-person training session (duration 2 h) with physicians on intervention delivery, including shared decision-making principles and S.M.A.R.T. (specific, measurable, attainable, realistic, time-based) goal-setting [28].

#### Toolkit and documentation (experimental group)

The toolkit includes CCAs as well as a CCA chart note and a goal-setting sheet (Additional file 2).

##### *Conversation Cards for Adolescents*©

**Fig. 1:**
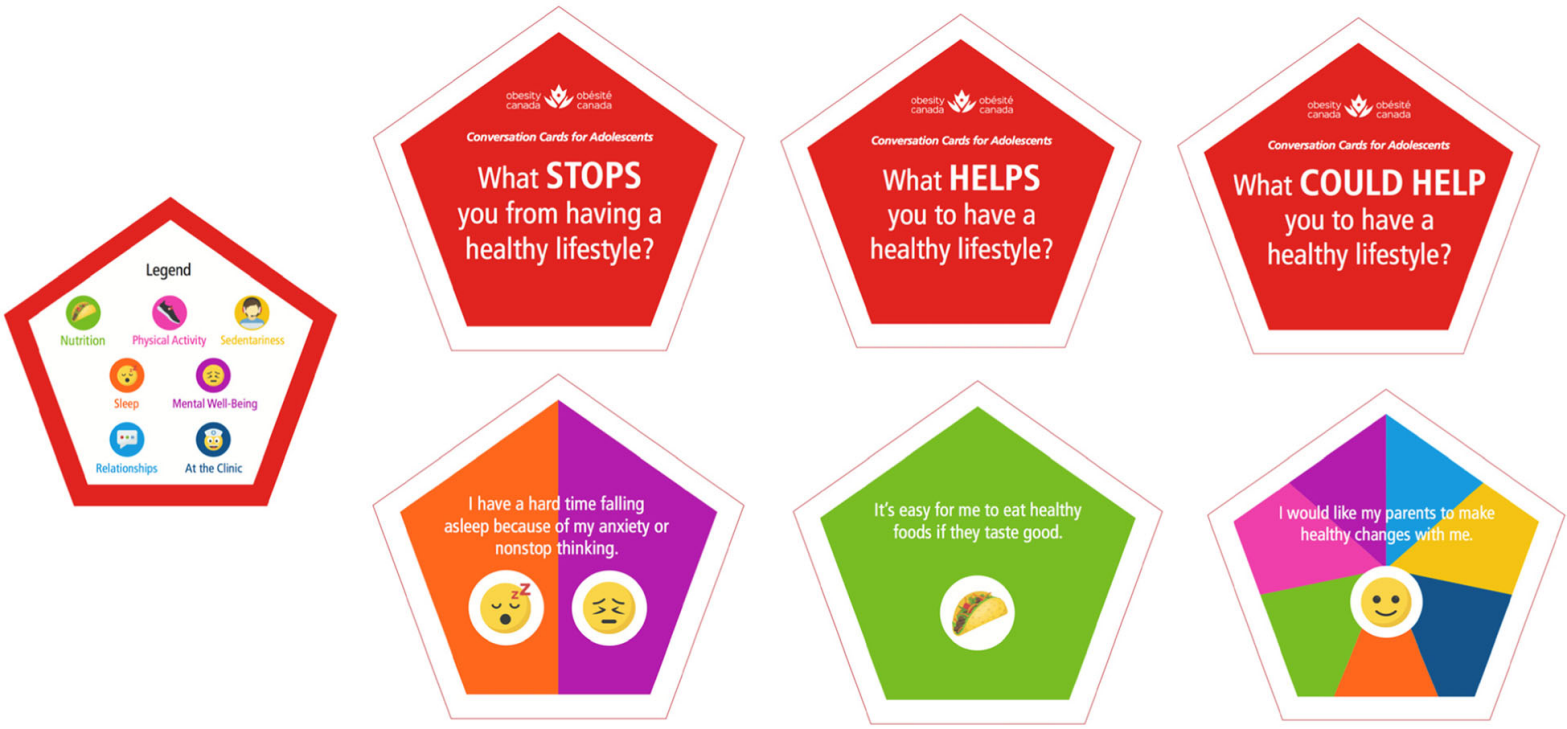
Example cards per category in *Conversation Cards for Adolescents*©

The development of CCAs is described elsewhere (Kebbe M, Perez A, Buchholz A, McHugh TLF, Scott SD, Richard C, Dyson MP, Ball GDC: Conversation cards for adolescents©: a communication and behavior change tool for health care providers and adolescents with obesity, submitted). Briefly, CCAs are a hard-copy deck of 45 cards that are organized into three categories, including: *What STOPS you from having a healthy lifestyle? What HELPS you to have a healthy lifestyle?* and *What COULD HELP you to have a healthy lifestyle?* Each category contains an individual statement pertaining to one or more of following suits: nutrition, physical activity, sedentariness, sleep, mental well-being, relationships, and clinical factors (please see Fig. 1 for CCA card examples). After the study coordinator provides them with instructions for completing the task, adolescents examine the deck of CCAs and independently select the top 3 factors that resonate most with them in changing their lifestyle habits.

##### CCA chart note

After completing the CCA activity, adolescents will record their top 3 choices on a chart note. Adolescents will leave the CCA deck with the study coordinator and bring the chart note with them into their clinical appointment. Following their appointment, the study coordinator will photograph the chart note using a study-specific iPad for documentation purposes. Adolescents will keep the hard-copy chart note with them as a frame of reference for the priorities of change they had identified.

##### Goal-setting sheet

Adolescents will be given a S.M.A.R.T. goal-setting sheet template to bring with them into their clinical appointment. Following the appointment, the study coordinator will photograph the goals sheet using the study-specific iPad for fidelity with S.M.A.R.T. goal-setting and documentation purposes. Adolescents will keep the hard-copy goal-setting sheet with them as a frame of reference for the changes they planned to make with their physician.

#### Administration

The entire administration process for both experimental and control groups, which will take place during scheduled clinical appointments after physicians address the primary health concern, should take no more than 25–30 min to perform. This is a 5–10-min addition to regular clinical visit length, which is to test the feasibility of the goal-setting process in a real-world, practice environment.

##### Experimental group

Adolescents randomized to the experimental group will be asked to complete the CCA activity in a quiet, private room at the clinic 15 min before their clinical appointment. Adolescents will review their choices (as indicated on the CCA chart note) with their physician, who will help them set a S.M.A.R.T. goal for their primary priority. Adolescents will be advised to make one goal, which will be documented on the goal-setting sheet, to ensure that achievement over our 3-week follow-up period is feasible.

##### Control group

Adolescents in the control group will attend their scheduled clinical appointment with their physician, including any education, information, or additional consultations as deemed necessary by their provider. Control group adolescents will not complete the CCA activity or any tool-related outcome measures; however, they will set one S.M.A.R.T. lifestyle goal with their provider.

In addition to collaborative goal-setting, physicians (SL, HR, TL, BI, LJ, FN, KT, JLW, MZ) will use shared decision-making principles with both groups to maximize patient-centered care. We will debrief with each physician after their first experimental and first control clinical appointments to solicit feedback, reinforce intervention fidelity, and make any necessary modifications to intervention procedures (please see Additional file 3 for the detailed intervention procedures for physicians).

### Demography, anthropometry, clinical, and intervention data

For efficiency, we will collect demographic data (e.g., date of birth, sex) verbally from adolescents. We will obtain measured height and weight data at the point of eligibility screening; existing equipment at the NECHC will be used to measure this data based on established protocols established [29]. We will also document clinical (e.g., physician name, primary health concern) and intervention-related (e.g., appointment duration, top 3 priorities) data. This information will be documented on a data file separate from a master file containing participants’ IDs and names. Physicians will complete a sociodemographic survey (hard-copy or electronic), which will document variables such as date of birth, year of graduation from terminal degree, and number of years in providing care to adolescents. We will document this information on a password-secure Excel spreadsheet and perform source document verification to ensure consistency between the data collected and entered.

### Psychometric data

#### Primary outcome

We will measure feasibility metrics and thresholds of success outlined in Table 1, as well as process, resource, management, and scientific assessments. These evaluations were informed by a modified version of the framework described by Tickle-Degnen (2013) [30] and will either have specific quantifiable thresholds or will be evaluated by the on-site study coordinator, physicians, and administrative/clinical staff through direct observation and experiences from the trial.

**Table 1:** Feasibility metrics and thresholds of success

Feasibility metric	Measure of success
Practicality	Time: • 15 min to complete tool activity in waiting room • No differences in appointment length (experimental vs control) • 15 min to complete outcome measures post-appointment Commitment: • Recruitment: Willingness of staff to help recruit adolescents; ≥ 85% recruited (“*n*” enrolled/“*n*” screened and eligible for enrollment) • Attrition: ≤ 15%
Implementation	≥ 95% completion of tool activity by adolescents ≥ 95% completion of goal-setting sheet by adolescents and physicians
Demand	Expressed interest or intention by clinic staff to use the tool post-study
Randomization protocol	• ≥ 85% adolescents willing to be randomized • 100% accuracy of randomization procedures
Measurement protocol	100% and ≥ 85% assessment completed by adolescents at baseline and 3-weeks follow-up, respectively

### Process assessment

1. What is the expected:
  - Number of eligible members of the targeted population?
  - Recruitment proportion?
  - Refusal proportion for participation and for randomization?
  - Retention and follow-up proportions as the participants move through the trial?
2. How feasible and suitable are:
  - Eligibility criteria? Are criteria clear and sufficient or too inclusive or restrictive?
  - Data collection assessments? Do participants understand the questions and other data collection methods? Do they respond with missing or unusable data?
  - Data collection procedures? Do the participants have enough time and capacity to complete data collection procedures? Does the overall data collection plan involve a reasonable amount of time, or does it create a burden for the participants?

### Resource assessment

1. Does the clinical environment have the:
  - Physical capacity to handle the number of participants (e.g., private room to complete CCAs)?
  - Time to conduct each stage and aspect of the protocol? What are the time frames, and how do they coordinate with other responsibilities? How long does it take to connect with a participant?
  - Equipment (e.g., CCAs, iPad) in the correct place at the correct time? What equipment is needed, and is it available when needed?
  - Ability to deal with broken, lost, or stolen equipment and materials? Are there backup plans for obtaining needed equipment and materials?
  - Adequate software to capture and use data? What software is available for conducting the research?
  - Clinical site’s willingness, motivation, and capacity to carry through with study-related tasks and to support researchers’ time and effort? What administrative services are in place for research at this level?
  - Documented evidence indicating that these centers abide by their commitments? What are the challenges in fulfilling research support commitments?
  - Access to services, such as printing, copying, and technology (e.g., WIFI for on-site randomization)?

### Management assessment

1. What are the challenges and strengths of:
  - The investigators’ administrative capacity to manage the planned RCT?
  - Research investigator and staff capacities, expertise, and availability for the planned research activities?
  - Formats and structures of forms that document participant progress through the trial?
  - Accurate data entry into the computer? Are data lost, forgotten, or entered incorrectly? How are data files organized, named, and dated? Who is in charge of tracking the latest data entry and the quality of entry?
  - Matching of data to participants from different sources (e.g., allocation group with corresponding outcome assessments)?
  - Management of the ethics of the research? To what extent do staff comply with the approved research protocol? How effectively are adverse events (e.g., feeling overwhelmed from working on lifestyle) during implementation identified, documented, and reported? What happens if a participant experiences a clinical emergency or if family abuse is identified during the trial?

### Scientific assessment

1. What is the level of safety of the procedures in the intervention or interventions?
2. What is the level of safety and burdensomeness of the frequency, intensity, and duration of the intervention? Can these and other elements be standardized in a protocol without loss of a patient-centered, individualized focus?
3. What are the reliability, validity, and trustworthiness of the assessments for the targeted population for this specific intervention? Do the assessments capture individual participants’ needs and measure their responsiveness to these needs?
4. What values constitute clinically meaningful differences on assessment procedures?
5. What is the expected degree of change (i.e., responsiveness) of the participants?
6. What are the estimates of the intervention effect and the variance of that effect across the planned population?

#### Secondary outcomes

We will use a range of questionnaires to examine behavioral, affective-cognitive, and process evaluation outcomes. Adolescents and physicians will be given the option to complete the instruments outlined below using paper-based copies or online using a study iPad. We selected several reliable and validated patient-reported outcome measures on collaborative goal-setting and tool user experiences, or researcher-developed questionnaires/interview guides on the degree of and effort made for goal achievement, outcome prioritization, and tool likeability, usefulness, feasibility, and usability. For physicians, we will explore tool user experiences, tool acceptance and adoption, tool likeability, usefulness, feasibility, and usability, and appointment duration. All physicians are exposed to the CCAs in the orientation and training sessions, as well as before completing the tool assessments. When different reliable and validated questionnaires on the same topic were available in the literature, we chose ones that were most relevant to our study and did not require any adaptations (please see Additional file 4 for the questionnaires and interview guide).

##### Technology acceptance model

This questionnaire uses a 7-point scale and includes 11 items representing perceived usefulness and perceived ease of use of a technology [31].

##### User experience questionnaire

This questionnaire uses a 7-point scale to represent participants’ agreement on 26 contrasting attributes that may apply to a product [32].

##### Tool likeability, usefulness, feasibility, and usability

To assess these constructs, we will use open- and closed-ended researcher-developed questions modified for both adolescents and physicians.

##### Patient perception of collaborative goal-setting

This questionnaire uses a 5-point scale and includes five factors: listen and learn from each other; share ideas; caring relationship; agree on a measurable objective; support for goal achievement [33].

##### Appointment duration

The study coordinator will time the duration of the clinical appointments for both experimental and control groups using a timer on the study-specific iPad.

##### Telephone interview

The study coordinator will call adolescents (duration ~ 30 min) to inquire about their participation (e.g., using a 0–9 scale researcher-developed questions for the degree of changes made to achieve the set goal) and engagement (e.g., study procedures, including ranking the outcome measures used for this study in order of importance).

### Schedule of assessments

There are three data collection time points, each of which will take 15–30 min to complete. At time zero (T0), measurements will occur immediately *before* the clinical appointment; at T1, measurements will occur immediately *after* the clinical appointment; and at T2, a follow-up assessment will take place 3 weeks after the scheduled clinical appointment. Outcome measures collected at the three time points for adolescents and physicians are indicated in Tables 2 and 3.

**Table 2:** Outcome measures and assessment time points for adolescents

Variables	Measure	Instrument	Assessment interval	
			Baseline	Follow-up
Primary outcome	Feasibility metrics and thresholds documented continually			
Secondary outcomes–behavioral and affective-cognitive outcomes	Collaborative goal-setting Degree of and effort made for goal achievement	Patient perception of collaborative goal setting Researcher-developed question	T1	T2
Secondary outcomes–process evaluation items	Tool user experience* Tool likeability, usefulness, feasibility, and usability* Priorities for outcome measures	User experience questionnaire Researcher-developed questionnaire Researcher-developed question	T0 T1	T2

*Experimental group only

**Table 3:** Outcome measures and assessment time points for physicians

Variable	Measure	Instrument	Assessment Interval	
			Baseline	Follow-up
Primary outcome		Feasibility metrics and thresholds documented continually.		
Secondary outcomes–process evaluation items	Tool user experience Tool acceptance and adoption Tool likeability, usefulness, feasibility, usability	User experience questionnaire Technology acceptance model Researcher-developed questionnaire	T0	T2 T2 T2

### Data analysis

Due to a lack of research studies directly comparable to ours, we were unable to use recommendations for pilot trial sample sizes on the basis of effect size for a future primary outcome on collaborative goal-setting [34]. We justified our pilot trial sample size of 50 adolescents with pragmatic considerations. That is, assuming ~ 85% recruitment of our sample of 50 adolescents, we estimated a margin of error of ± 10% for a 95% confidence interval; this recruitment percentage is derived from similar RCTs conducted in a primary care setting related to pediatric obesity [35, 36].

As per recommendations for pilot studies, our analysis will be primarily of a descriptive nature on feasibility outcomes [37]. Adolescent and physician characteristics will be summarized using descriptive statistics. Proportions, between-group differences, and correlations for quantitative secondary outcome measures completed by adolescents will be examined using descriptive statistics, independent sample *T* tests, and regression analyses respectively, as conducted by the blinded Data Coordinating Centre statistician from the Women and Children’s Health Research Institute (University of Alberta). Contingent on sample size distributions by weight, we will also examine potential differences in responses among adolescents of regular-weight versus those with overweight, obesity, or severe obesity; the analysis will adhere to the intention-to-treat principle in that none of the enrolled (randomized) adolescents will be excluded from the analysis and all patients will be analyzed according to the randomization scheme. Qualitative data will be audio-recorded, transcribed verbatim using *The Comma Police*, managed using *NVivo 11*, and analyzed by two independent reviewers using content analysis [38]; field notes and memos will be documented.

### Evaluation of implementation

We will use the Centers for Disease Control and Prevention Framework for Program Evaluation for a qualitative evaluation of our intervention [39]. This framework is suitable and relevant to how physicians practice clinically, and includes engaging stakeholders, describing the interventions, focusing the evaluation design, gathering credible evidence, justifying conclusions, and ensuring use and sharing lessons learned. This evaluation is not linear; however, earlier steps (e.g., engaging physicians in the design of the trial) provide the foundation for subsequent steps (e.g., ensuring the trial procedures are clinically relevant). The purpose of this qualitative evaluation is to understand the impact and implementation of our interventions (e.g., advantages, disadvantages) and facilitate its integration and sustainability at the NECHC and similar health care settings (e.g., by identifying barriers and enablers). Program evaluations are best completed in a team approach; we will plan an end-of-study evaluation team meeting (~ 1 h) to discuss feasibility metrics between the research team, physicians, and administrative/clinical staff.

### Project management considerations

#### Data management

We will use Microsoft® Excel and REDCap®, a secure, online data collection and management platform. REDCap® is hosted and supported by the Women and Children’s Health Research Institute (University of Alberta).

#### Clinical trial registration

We registered our trial on clinicaltrials.gov (Identifier: NCT03821896) prior to patient recruitment and iteratively make note of any prospective changes to our study. Protocol amendments will be made on an as-needed basis.

#### Research ethics considerations

We will obtain ethics approval from the Human Research Ethics Board (University of Alberta) and operational approval from Alberta Health Services (Edmonton, Alberta, Canada). Participants may experience psychosocial adverse events in relation to making lifestyle changes, which will be recorded and monitored by the on-site research, administrative, or clinical staff. All adolescents, parents, and physicians enrolled in our trial will provide written informed assent (adolescents) and/or consent (parents and physicians).

#### Confidentiality

Confidentiality will be explained to participants as part of the consent process. We will only collect personal health information relevant for this study. To protect identity, participants will be assigned a number and names and other identifying information will be removed for analysis. Further, any information shared outside of our research team will be done at the group-level, so no individuals will be named or identified. Any information on a computer will be protected with a password and saved for 5 years on a secure server maintained by MedIT (Faculty of Medicine & Dentistry, University of Alberta). Hard-copy documentation will also be securely stored for 5 years at the Edmonton Clinic Health Academy (University of Alberta) in a locked filing cabinet.

#### Knowledge translation, exchange, and dissemination

Our primary aim in evaluating the feasibility of CCAs in our pilot trial is to ultimately improve adolescent-provider communication related to shared decision-making and goal-setting for preventing or managing pediatric obesity. We will use a collaborative approach of integrated knowledge translation [40] to engage our end-users (adolescents, physicians) throughout the study; this will ensure that outputs are relevant and practice to specific audiences [40–42]. For example, in qualitatively exploring adolescents’ priorities in the context of their clinical encounter as well as physicians’ study conceptualizations and evaluations on the intervention implementation, we can help ensure future applications of CCAs are relevant and actionable in the practice setting. In addition to disseminating our findings via peer-reviewed publications and conference presentations, we plan to create infographics detailing the development and evaluation of CCAs; these will be shared internally with team members and our research participants as well as externally through various social media platforms (e.g., study blog: http://www.teensaid.wordpress.com).

## Discussion

A complex set of biological, social, and environmental factors contribute to the high prevalence of obesity in children and adolescents. The complexity of obesity is especially apparent in adolescents who undergo a number of physical, physiological, and psychosocial changes as they grow and develop. Early-intervention settings currently lack novel and developmentally appropriate tools for providers to prevent and manage adolescent obesity, with existing lifestyle interventions only showing minimal effectiveness. With this in mind, we have a compelling case for developing and evaluating our clinical, bilingual tool as a means of targeting and tailoring obesity prevention and management approaches among adolescents.

Our research will contribute to real-world clinical settings for obesity prevention and management while emphasizing patient-centered care. Patient-centered care may decrease health care costs since patients play a more active role in their own health care to collaboratively reach a correct diagnosis and create personalized treatment plans with their providers. For example, a randomized study by Bertakis and Azari (2011) showed that a higher average amount of patient-centered primary care visits was associated with significant decreases in the annual number of specialty-care visits, hospitalizations and diagnostic services, and laboratory and diagnostic test charges [43]. This approach to health care decision-making also fosters the invaluable role that adolescents are encouraged to assume in their own health care. In doing so, it recognizes increasing adolescent autonomy and posits that adolescents present with unique experiences, needs, and priorities that may not otherwise be captured in traditional models of care. Particularly, using S.M.A.R.T. goal-setting is consistent with recommendations outlined in a review of childhood obesity by Kumar and Kelly (2017) [44]. Because adolescents often set unrealistic goals [45], S.M.A.R.T. goals can be an effective way in which providers encourage adolescents to strive for healthy lifestyle changes. S.M.A.R.T. is especially relevant to short- vs long-term goals, and is consistent with our chosen 3-week follow-up period.

We chose the NECHC based on expressed interest from on-site providers in expanding their scope of practice beyond clinical care exclusively to include clinical and health services research that aligns with their approach to pediatric and family health. During our study design phase, we undertook consultations with relevant stakeholders, including physicians, nurses, administrative/clinical staff, and researchers. This was to ensure that the study design and procedures were acceptable to all parties involved, for example, by ensuring relevance to the way in which physicians deliver their clinical services to further support study implementation.

In addition to examining experiences in using CCAs, our trial will support assessment and decision-making for a future full-scale RCT. Examining feasibility as the primary outcome of this study will inform necessary modifications in regards to the full study design and procedures. The planning, conduct, and reporting of our research is in alignment with a recent *Pediatric Obesity* issue showcasing and emphasizing the importance of novel and methodologically rigorous RCTs focused on preventing and managing pediatric obesity [46]. The progression of this pilot RCT to an effectiveness RCT will provide an evidence base of an approach to manage adolescent obesity that is appealing to diverse stakeholders, including physicians, adolescents, and caregivers. If effective, CCAs will offer benefits in adolescent-provider communication, adolescent experiences in care, and improved lifestyle habits among adolescents.

## Supplementary information

**Additional file 1.** SPIRIT 2013 Checklist: Recommended items to address in a clinical trial protocol and related documents. SPIRIT checklist

**Additional file 2 ***Conversation Cards for Adolescents*© toolkit. CCA chart note and goal-setting sheet

**Additional file 3.** Intervention procedures for physicians. Intervention procedures (experimental and control) for physicians

**Additional file 4.** Outcome measure instruments and interview guides. Quantitative and qualitative data collection instruments

## Data Availability

Not applicable.
