# Supplementary material for: Feasibility, user experiences, and preliminary effect of Conversation Cards for Adolescents© on collaborative goal-setting and behavior change: protocol for a pilot randomized controlled trial
Source: Pilot Feasibility Stud. 2019 Dec 18;5:149. doi: 10.1186/s40814-019-0533-3 (PMC6918564; doi:10.1186/s40814-019-0533-3)
Supplement: Supplementary file 2 — Additional file 2 Conversation Cards for Adolescents© toolkit. CCA chart note and goal-setting sheet [file 40814_2019_533_MOESM2_ESM.pdf]

Date (dd/mm/yyyy): ______ /_______ /__________

Name: ____

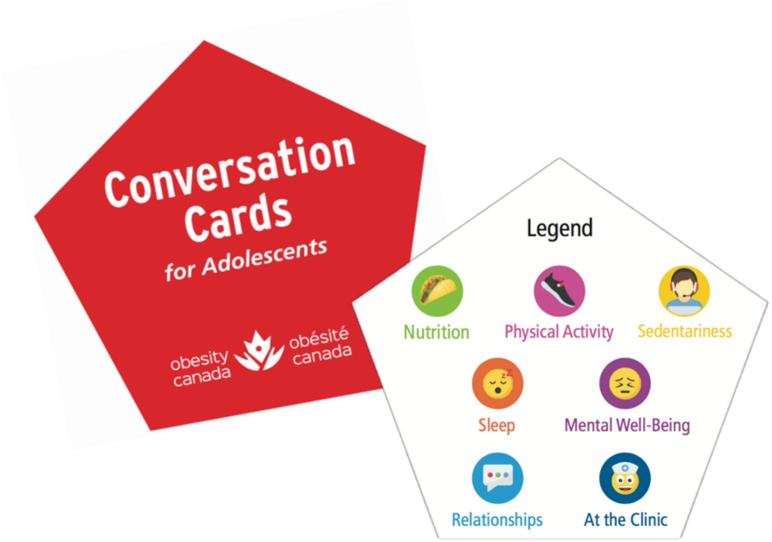

Please insert a check mark near your TOP priorities.

What STOPS you from having a healthy lifestyle?

____ *I enjoy using technology, especially when I'm bored.*

____ *My parents are on my case about my eating habits.*

____ *It's hard for me to be active at the end of the day when I'm tired.*

____ *I feel like I'm being watched or judged when doing physical activity in public.*

____ *It's hard to get back on track when I haven't been active for a while.*

____ *I tend to choose technology over being active (examples: gaming, social media).*

____ *My parents tend to take over the conversation during appointments with my clinicians.*

____ *My parents feel the need to fix everything.*

____ *Unhealthy foods get especially tempting during special occasions and holidays.*

____ *I'm rewarded with unhealthy food on some occasions.*

____ *I feel like I have no control over my sleep (example: how fast to fall asleep).*

____ *My parents and I have different priorities.*

____ *I have a hard time falling asleep because of my anxiety or nonstop thinking.*

____ *Sometimes my weight makes me feel like I don't fit in.*

____ *I have nothing else to do, so I go online or play video games.*

What HELPS you to have a healthy lifestyle?

____ *It's easier for me to be active when I genuinely enjoy the activity.*

____ *It's easier to be active with people I know.*

____ *It’s easy for me to eat healthy foods if they taste good.*

____ *We have enough money to afford healthy foods.*

____ *It's helpful to start small and gradually work up when making lifestyle changes.*

____ *I’m committed to losing weight to look better.*

____ *It helps that my friends believe in me.*

____ *I feel energized after being active.*

____ *Some activities help me to relieve stress.*

____ *Having a ride to my activities helps me be active.*

____ *I’m more inclined to be active when the weather is nice.*

____ *I’m more likely to be active when someone is motivating me.*

____ *It helps when I have someone be active with me.*

____ *I fall asleep quickly after a long day.*

____ *Talking with my friends (online or in-person), family, or clinicians helps with my anxiety or depression.*

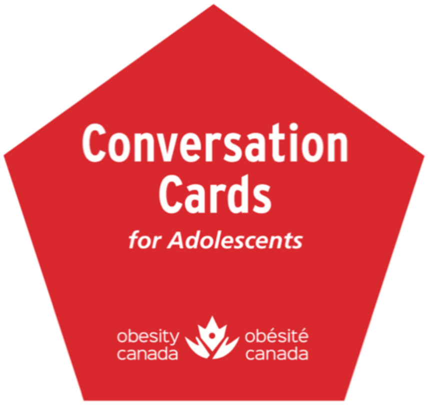

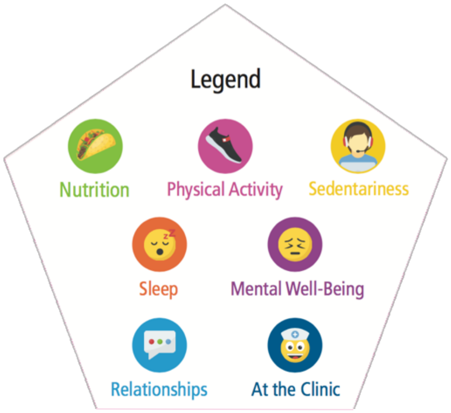

Last Updated: December 14, 2018

What COULD HELP you to have a healthy lifestyle?

____ *I would like taxes to be removed from healthy foods.*

____ *I would like school to start later so I can get more sleep.*

____ *I value privacy between myself and my clinician.*

____ *It's my body, so I should make the final decision about my treatment plan.*

____ *I would like my parents to make healthy changes with me.*

____ *I would like physical activity programs to be better advertised so I know what's available.*

____ *I would like packaging of unhealthy food to not look so fancy and appealing.*

____ *I would like my parents to stop criticizing and judging me.*

____ *I would like to be able to bring food into class.*

____ *I would like to have kids my age in my neighborhood to be active with.*

____ *I would like my dietitian to share healthy recipe ideas with my family.*

____ *I want to have more control over my clinical appointments than my parents (examples: talk more, be involved in decision-making).*

____ *I would like more varied physical activity options in my community.*

____ *I'm old enough to come to my clinical appointments without my parents.*

____ *I would like there to be more healthy foods at my home.*

Conversation Cards
for Adolescents
obesity canada obésité canada

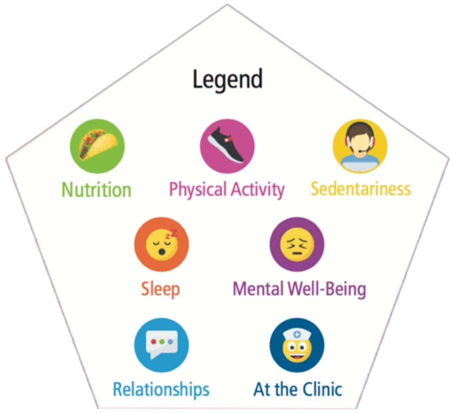

Last Updated: December 14, 2018

Notes / Follow-Up Plan:

Frequently Asked Questions:

How can I help a teen who selected cards beyond my area of comfort or expertise?

Given the range of issues included, it would be surprising if you felt confident and competent in all areas. Along with validating teens' experiences, as with other topics that emerge in your clinical interactions that are beyond your expertise, it is completely reasonable to seek support and information from a colleague(s) to assist you or to refer your patient to a clinician or service so they can receive the support they seek.

What if teens aren't comfortable selecting some cards in the presence of their parents?

As with any clinical appointments with teens, it is important for them to know your role and how you can best support them. Sometimes, this may include meeting with them individually, without their parents; however, when teens are ready, willing, and able to make healthy lifestyle and behavioural changes, the support of their parents or other important adults in their lives is very important in helping them to make and maintain healthful changes.

What if teens select cards that do not align with their parents' priorities?

Conversation Cards for Adolescents were created for and with teens, so their priorities may differ from those of their parents. Ideally, areas of focus that both teens and parents can work on as a family may lead to the greatest likelihood of success. In the event that agreement can't be achieved, your skill and experience as a clinician are important to help reconcile differences of opinion, along with identifying ways for parents to support their teens even when they disagree about their priorities.

INTERVENTION TECHNIQUES

Conversation Cards for Adolescents – A Pilot RCT

Decision-Making Principles:

1. Develop a partnership with adolescents
2. Review adolescents' preference for role in decision-making
3. Explore and respond to adolescents' ideas, concerns, and expectations
4. Identify/discuss choices (top 3 priorities selected)
5. Make or negotiate a decision (top 1 of 3 priorities selected) with the adolescent
6. Agree on an action plan (set S.M.A.R.T. goal collaboratively – *see below*)

S.M.A.R.T. Goals:

Specific Goals: Set specific objectives and goals, such as establishing a start date or agreeing on an explicit behavior change step.

Measurable Goals: Weight and behavior change goals should be measurable, such as aiming for a 10-minute walk after lunch on weekdays.

Achievable Goals: Over time, unrealistic goals can reduce motivation. Achievable weight loss goals, such as aiming to lower calorie intake by 300 calories per day, rather than overly restrictive diet goals, may improve success.

Relevant Goals: Goals should align with priorities. For one adolescent, losing weight to help her move more or better is meaningful; another adolescent may be more energized after exercising with a friend.

Time-sensitive Goals: A mutually-agreed upon timeline for achieving a specific goal helps motivation. Ask adolescents – What is reasonable to achieve today? This week? This month?

Let's make "I will exercise more this year" into a S.M.A.R.T. goal.

1. **Specific** – The activity I want to do is running. Because of the cold weather, I will go to my nearest gym.

2. **Measurable** – I will run for 15 minutes on Tuesdays, Thursdays, and Saturdays.

3. **Achievable** – Since I haven't ran in a while, I will start slow and gradually increase the duration over the indicated time period.

4. **Relevance** – I prefer cardio to weights, so I will choose running as my activity.

5. **Time**-bound – I will start this goal on Tuesday, March 5th, and re-evaluate it in 3 weeks to see if I need to increase or decrease the amount of time running or the frequency of runs to make sure my resolution remains possible and effective.

NEW GOAL

"Starting March 5th, I will run for 15 minutes 3 times each week (Tues, Thurs, Sat) for the next 3 weeks at my nearest gym."

	1	2	3	4	5	6
	**WAYS I CAN IMPROVE MY LIFESTYLE – WHAT?**	**WHAT WILL STOP YOU?**	**HOW MUCH?**	**HOW OFTEN?**	**WHEN?**	**WHERE?**
**SET YOUR GOAL**	Be more physically active<br><br>Activity: Running	Weather	15 minutes per day	Three times per week	Tuesdays, Thursdays, and Saturdays<br><br>Starting March 5th	At my nearest gym
**FINAL**	Starting March 5th, I will run for 15 minutes 3 times each week (Tues, Thurs, Sat) for the next 3 weeks at my nearest gym.					

	1	2	3	4	5	6
	WAYS I CAN IMPROVE MY LIFESTYLE – WHAT?	WHAT WILL STOP YOU?	HOW MUCH?	HOW OFTEN?	WHEN?	WHERE?
SET YOUR GOAL						
FINAL						
