# Supplementary material for: Feasibility, user experiences, and preliminary effect of Conversation Cards for Adolescents© on collaborative goal-setting and behavior change: protocol for a pilot randomized controlled trial
Source: Pilot Feasibility Stud. 2019 Dec 18;5:149. doi: 10.1186/s40814-019-0533-3 (PMC6918564; doi:10.1186/s40814-019-0533-3)
Supplement: Supplementary file 3 — Additional file 3. Intervention procedures for physicians. Intervention procedures (experimental and control) for physicians [file 40814_2019_533_MOESM3_ESM.pdf]

INTERVENTION PROCEDURES

Conversation Cards for Adolescents – A Pilot RCT

After teens are informed of the study, complete the informed consent forms, get randomly assigned to 1 of 2 groups (experimental *vs* control), and complete some study processes, pediatricians' interactions with teens enrolled in the study will include:

1. Experimental Group (n=25 teens; estimated time: 25-30 min/teen)

Teens will be given a goal-setting sheet &
a Conversation Cards chart note before their appointment

STEP 1 – Acknowledge the research study

Suggested wording: "*I see you agreed to participate in the research study and you completed the activity with the Conversation Cards. Thanks for participating! We'll go over the next part of the study together, but first, let's talk about why you're here to see me today.*"

STEP 2 – Discuss and address primary health concern for visit

STEP 3 – Briefly describe the research study

Suggested wording: "*OK, back to the Conversation Cards. As Maryam explained before, the cards were created for teens like you to help them make healthy lifestyle changes. As you know, it's important for teens to eat healthy, be physically active, and get enough sleep. These are some of the topics included in the cards.*"

STEP 4 – Complete the Conversation Card task and set one S.M.A.R.T. goal

Suggested wording:

1. *"What did you think of the cards?"*
2. *"What were your top 3 choices ***MD to refer to chart note***? Why did you choose these?"*

3. *“And of these 3, which is the one that you would most want to work on?”*
4. *“Since this issue was most important to you, I’d like to work with you to set a goal related to this issue.”*
    a. *“What kind of goal do you think would help you with this issue?”* (***MD to refer to S.M.A.R.T. goal-setting sheet to discuss and finalize the goal***)

STEP 5 – Discuss next steps

Suggested wording: *“You can keep this goal-setting sheet with you to help you remember your goal for the next 3 weeks. Maryam should be waiting for you in the waiting room with some surveys to complete before you go and the next steps for the study. It was great seeing you today!”*

2. Control Group (n=25 teens; estimated time: 25-30 min/teen)

Teens will be given a goal-setting sheet before their appointment.

STEP 1 – Acknowledge the research study

Suggested wording: "*I see you agreed to participate in the research study. Thanks for participating! We'll go over the next part of the study together, but first, let's talk about why you're here to see me today."*

STEP 2 – Discuss and address primary health concern for visit

STEP 3 – Briefly describe the research study

Suggested wording: *"OK, back to the research study. As Maryam likely mentioned before, it's very important for all teens to eat healthfully, be physically active, and get enough sleep."*

STEP 4 – Set S.M.A.R.T. goal

Suggested wording: *"Let's set a goal for you to work on over the next 3 weeks."*

a. *"Is there a specific part of your lifestyle that you would want to work on related to food, exercise, sleep or something else?"*
b. *"What kind of goal do you think would help you with this issue?"* (***MD to refer to S.M.A.R.T. goal-setting sheet to discuss and finalize the goal***)

STEP 5 – Discuss next steps

Suggested wording: *"You can keep this goal-setting sheet with you to help you remember your goal for the next 3 weeks. Maryam should be waiting for you in the waiting room with some surveys to complete before you go and the next steps for the study. It was great seeing you today!"*
