# Supplementary material for: Feasibility, user experiences, and preliminary effect of Conversation Cards for Adolescents© on collaborative goal-setting and behavior change: protocol for a pilot randomized controlled trial
Source: Pilot Feasibility Stud. 2019 Dec 18;5:149. doi: 10.1186/s40814-019-0533-3 (PMC6918564; doi:10.1186/s40814-019-0533-3)
Supplement: Supplementary file 4 — Additional file 4. Outcome measure instruments and interview guides. Quantitative and qualitative data collection instruments [file 40814_2019_533_MOESM4_ESM.pdf]

User Experience Questionnaire (Providers)

Please make your evaluation now.

For the assessment of *Conversation Cards for Adolescents* (CCAs), please fill out the following questionnaire. The questionnaire consists of pairs of contrasting attributes that may apply to the product. The circles between the attributes represent gradations between the opposites. You can express your agreement with the attributes by ticking the circle that most closely reflects your impression.

<u>Example:</u>

Attractive	▫	▪	▫	▫	▫	▫	Unattractive

This response would mean that you rate CCAs as more attractive than unattractive.

Please decide spontaneously. Don’t think too long about your decision to make sure that you convey your original impression.

Sometimes you may not be completely sure about your agreement with a particular attribute or you may find that the attribute does not apply completely to the particular product. Nevertheless, please tick a circle in every line.

It is your personal opinion that counts. Please remember: there is no wrong or right answer!

	1	2	3	4	5	6	7		
annoying	○	○	○	○	○	○	○	enjoyable	1
not understandable	○	○	○	○	○	○	○	understandable	2
creative	○	○	○	○	○	○	○	dull	3
easy to learn	○	○	○	○	○	○	○	difficult to learn	4
valuable	○	○	○	○	○	○	○	inferior	5
boring	○	○	○	○	○	○	○	exciting	6
not interesting	○	○	○	○	○	○	○	interesting	7
unpredictable	○	○	○	○	○	○	○	predictable	8
fast	○	○	○	○	○	○	○	slow	9
inventive	○	○	○	○	○	○	○	conventional	10
obstructive	○	○	○	○	○	○	○	supportive	11
good	○	○	○	○	○	○	○	bad	12
complicated	○	○	○	○	○	○	○	easy	13
unlikable	○	○	○	○	○	○	○	pleasing	14
usual	○	○	○	○	○	○	○	leading edge	15
unpleasant	○	○	○	○	○	○	○	pleasant	16
secure	○	○	○	○	○	○	○	not secure	17
motivating	○	○	○	○	○	○	○	demotivating	18
meets expectations	○	○	○	○	○	○	○	does not meet expectations	19
inefficient	○	○	○	○	○	○	○	efficient	20
clear	○	○	○	○	○	○	○	confusing	21
impractical	○	○	○	○	○	○	○	practical	22
organized	○	○	○	○	○	○	○	cluttered	23
attractive	○	○	○	○	○	○	○	unattractive	24
friendly	○	○	○	○	○	○	○	unfriendly	25
conservative	○	○	○	○	○	○	○	innovative	26

Evaluation Questionnaire (Providers)

The following questions ask about your experience in using Conversation Cards for Adolescents (CCAs).

I. Likeability

1. I was **satisfied** with using CCAs.

☐ Strongly disagree ☐ Disagree ☐ Somewhat disagree ☐ Neutral ☐ Somewhat agree ☐ Agree ☐ Strongly agree

2. CCAs were **relevant** for me to use in my clinical consultations.

☐ Strongly disagree ☐ Disagree ☐ Somewhat disagree ☐ Neutral ☐ Somewhat agree ☐ Agree ☐ Strongly agree

3. What did you **like** the most about CCAs?

____

____

____

4. What did you **dislike** the most about CCAs?

____

____

____

5. Are there too many cards, not enough, or just the right amount?

____

____

____

II. Usefulness

1. CCAs **improved** my rapport with my adolescent patients.

☐ Strongly disagree ☐ Disagree ☐ Somewhat disagree ☐ Neutral ☐ Somewhat agree ☐ Agree ☐ Strongly agree

2. CCAs **improved** my communication with my adolescent patients.

☐ Strongly disagree ☐ Disagree ☐ Somewhat disagree ☐ Neutral ☐ Somewhat agree ☐ Agree ☐ Strongly agree

3. CCAs **helped** me to involve my adolescent patients in making decisions about their health and/or weight.

☐ Strongly disagree ☐ Disagree ☐ Somewhat disagree ☐ Neutral ☐ Somewhat agree ☐ Agree ☐ Strongly agree

4. In what other way(s) were CCAs **helpful**?

____

____

____

III. Feasibility

1. Adolescents had enough **time** to complete the CCAs activity before their appointment.

 Strongly disagree Disagree Somewhat disagree Neutral Somewhat agree Agree Strongly agree

2. I had enough **time** to incorporate CCAs into my clinical consultations.

 Strongly disagree Disagree Somewhat disagree Neutral Somewhat agree Agree Strongly agree

3. I had enough **time** to complete the goal-setting activity during my scheduled appointments.

 Strongly disagree Disagree Somewhat disagree Neutral Somewhat agree Agree Strongly agree

IV. Usability

1. I **intend to use** CCAs again in my future appointments.

 Strongly disagree Disagree Somewhat disagree Neutral Somewhat agree Agree Strongly agree

2. I would have to **change my behavior** significantly to attain the potential benefits of CCAs.

 Strongly disagree Disagree Somewhat disagree Neutral Somewhat agree Agree Strongly agree

3. Using CCAs would allow me to do things that I can't **easily do** now.

 Strongly disagree Disagree Somewhat disagree Neutral Somewhat agree Agree Strongly agree

4. I would **recommend** other *providers* to use CCAs with their adolescent patients.

☐ Strongly disagree ☐ Disagree ☐ Somewhat disagree ☐ Neutral ☐ Somewhat agree ☐ Agree ☐ Strongly agree

5. I would **recommend** *adolescents* to use CCAs.

☐ Strongly disagree ☐ Disagree ☐ Somewhat disagree ☐ Neutral ☐ Somewhat agree ☐ Agree ☐ Strongly agree

6. Do you have any feedback on how CCAs are **used**?

____

____

____

Technology Acceptance Model

Please rate each statement using the scale shown below (please select one response per row).

1. Using *Conversation Cards for Adolescents* in my job would enable me to accomplish tasks more quickly.

☐ Extremely likely ☐ Quite likely ☐ Slightly likely ☐ Neither ☐ Slightly unlikely ☐ Quite unlikely ☐ Extremely unlikely

2. Using *Conversation Cards for Adolescents* would improve my job performance.

☐ Extremely likely ☐ Quite likely ☐ Slightly likely ☐ Neither ☐ Slightly unlikely ☐ Quite unlikely ☐ Extremely unlikely

3. Using *Conversation Cards for Adolescents* would enhance my effectiveness on the job.

☐ Extremely likely ☐ Quite likely ☐ Slightly likely ☐ Neither ☐ Slightly unlikely ☐ Quite unlikely ☐ Extremely unlikely

4. Using *Conversation Cards for Adolescents* would make it easier to do my job.

☐ Extremely likely ☐ Quite likely ☐ Slightly likely ☐ Neither ☐ Slightly unlikely ☐ Quite unlikely ☐ Extremely unlikely

5. I would find *Conversation Cards for Adolescents* useful in my job.

☐ Extremely likely ☐ Quite likely ☐ Slightly likely ☐ Neither ☐ Slightly unlikely ☐ Quite unlikely ☐ Extremely unlikely

6. Learning to operate *Conversation Cards for Adolescents* would be easy for me.

☐ Extremely likely ☐ Quite likely ☐ Slightly likely ☐ Neither ☐ Slightly unlikely ☐ Quite unlikely ☐ Extremely unlikely

7. I would find it easy to get *Conversation Cards for Adolescents* to do what I want it to do.

☐ Extremely likely ☐ Quite likely ☐ Slightly likely ☐ Neither ☐ Slightly unlikely ☐ Quite unlikely ☐ Extremely unlikely

8. My interaction with *Conversation Cards for Adolescents* would be clear and understandable.

☐ Extremely likely ☐ Quite likely ☐ Slightly likely ☐ Neither ☐ Slightly unlikely ☐ Quite unlikely ☐ Extremely unlikely

9. I would find *Conversation Cards for Adolescents* to be flexible to interact with.

☐ Extremely likely ☐ Quite likely ☐ Slightly likely ☐ Neither ☐ Slightly unlikely ☐ Quite unlikely ☐ Extremely unlikely

10. It would be easy for me to become skillful at using *Conversation Cards for Adolescents.*

☐ Extremely likely ☐ Quite likely ☐ Slightly likely ☐ Neither ☐ Slightly unlikely ☐ Quite unlikely ☐ Extremely unlikely

11. I would find *Conversation Cards for Adolescents* easy to use.

☐ Extremely likely ☐ Quite likely ☐ Slightly likely ☐ Neither ☐ Slightly unlikely ☐ Quite unlikely ☐ Extremely unlikely

User Experience Questionnaire (Adolescents)

This survey was designed to examine how people think about *Conversation Cards for Adolescents (CCAs)*. The questionnaire includes opposing words that may apply to CCAs. The circles between the words represent the range for what you think of CCAs. For each pair of words, please ticking the circle that most closely reflects your impression of the cards.

<u>Example:</u>

Attractive	▫	▪	▫	▫	▫	▫	Unattractive

This answer means that you think CCAs are more ‘attractive’ than ‘unattractive’.

When you complete the survey, don’t think too long about your answers. We want to know your first impression, even if you aren’t 100% sure about your answers. There are no right or wrong answers!

	1	2	3	4	5	6	7		
annoying	○	○	○	○	○	○	○	enjoyable	1
not understandable	○	○	○	○	○	○	○	understandable	2
creative	○	○	○	○	○	○	○	dull	3
easy to learn	○	○	○	○	○	○	○	difficult to learn	4
valuable	○	○	○	○	○	○	○	inferior	5
boring	○	○	○	○	○	○	○	exciting	6
not interesting	○	○	○	○	○	○	○	interesting	7
unpredictable	○	○	○	○	○	○	○	predictable	8
fast	○	○	○	○	○	○	○	slow	9
inventive	○	○	○	○	○	○	○	conventional	10
obstructive	○	○	○	○	○	○	○	supportive	11
good	○	○	○	○	○	○	○	bad	12
complicated	○	○	○	○	○	○	○	easy	13
unlikable	○	○	○	○	○	○	○	pleasing	14
usual	○	○	○	○	○	○	○	leading edge	15
unpleasant	○	○	○	○	○	○	○	pleasant	16
secure	○	○	○	○	○	○	○	not secure	17
motivating	○	○	○	○	○	○	○	demotivating	18
meets expectations	○	○	○	○	○	○	○	does not meet expectations	19
inefficient	○	○	○	○	○	○	○	efficient	20
clear	○	○	○	○	○	○	○	confusing	21
impractical	○	○	○	○	○	○	○	practical	22
organized	○	○	○	○	○	○	○	cluttered	23
attractive	○	○	○	○	○	○	○	unattractive	24
friendly	○	○	○	○	○	○	○	unfriendly	25
conservative	○	○	○	○	○	○	○	innovative	26

Patient Perception of Collaborative Goal Setting (Adolescents)

Please rate each statement using the scale shown below (please select one response per row).

5-point scale**:** strongly disagree (1) – disagree (2) – neutral (3) – agree (4) – strongly agree (5)

Factor 1: Listen and learn from each other

1. I asked my doctor any questions I had
2. My doctor asked me if I had any concerns
3. My doctor explained the reasons for the goal
4. I learnt important things from my doctor
5. My doctor and I discussed the reasons for the goal
6. I listened to what my doctor had to say
7. I told my doctor important things about me
8. I told my doctor about any concerns I had
9. My doctor gave me the opportunity to ask any questions I had

Factor 2: Share ideas

10. I made sure my doctor knew about things that were important to me
11. I told my doctor about important things in my life
12. My doctor shared his/her ideas with me
13. I was interested in my doctor's ideas
14. My doctor provided important medical information to me
15. I shared my ideas with my doctor
16. I felt confident my doctor understood what was important to me

Factor 3: Caring relationship

17. My doctor treated me as a person
18. I respected my doctor's opinions
19. My doctor showed he/she cared about me as a person
20. My doctor respected my opinion
21. My doctor was honest with me
22. My doctor spent enough time with me
23. I showed my doctor that I care about achieving the goal
24. I was honest with my doctor

Factor 4: Agree on a measurable objective

25. I felt good about the goal
26. My doctor helped me understand what the specific goal is
27. I had confidence that I could achieve the goal
28. My doctor and I agreed on the specific goal that was set
29. My doctor and I discussed the potential specifics of the goal

Factor 5: Support for goal achievement

30. My doctor gave me information I could take home about the goal
31. I told my doctor and I discussed strategies for achieving the goal
32. I was comfortable discussing any challenges I might have achieving the goal
33. My doctor made me feel like I could achieve the goal
34. My doctor and I came up with a strategy for how to achieve the goal
35. My doctor checked to make sure I understood the goal
36. My doctor described how to achieve the goal

Evaluation Questionnaire (Teens)

The following questions ask about your experience using Conversation Cards for Adolescents (CCAs).

I. Likeability

1. I was **satisfied** with using CCAs.

☐ Strongly disagree ☐ Disagree ☐ Somewhat disagree ☐ Neutral ☐ Somewhat agree ☐ Agree ☐ Strongly agree

2. CCAs were **relevant** for me to use in the clinic.

☐ Strongly disagree ☐ Disagree ☐ Somewhat disagree ☐ Neutral ☐ Somewhat agree ☐ Agree ☐ Strongly agree

3. What did you **like** the most about CCAs?

____

____

____

4. What did you **dislike** the most about CCAs?

____

____

____

5. Are there too many cards, not enough, or just the right amount?

____

____

____

II. Usefulness

1. CCAs **improved** my rapport with my clinician.

☐ Strongly disagree ☐ Disagree ☐ Somewhat disagree ☐ Neutral ☐ Somewhat agree ☐ Agree ☐ Strongly agree

2. CCAs **improved** my communication with my clinician.

☐ Strongly disagree ☐ Disagree ☐ Somewhat disagree ☐ Neutral ☐ Somewhat agree ☐ Agree ☐ Strongly agree

3. CCAs **helped** me to make decisions about my health and/or weight with my clinician.

☐ Strongly disagree ☐ Disagree ☐ Somewhat disagree ☐ Neutral ☐ Somewhat agree ☐ Agree ☐ Strongly agree

4. CCAs **helped** me to take an active role in managing my health and/or weight.

☐ Strongly disagree ☐ Disagree ☐ Somewhat disagree ☐ Neutral ☐ Somewhat agree ☐ Agree ☐ Strongly agree

5. CCAs **helped** me to identify different factors that influenced my lifestyle habits.

☐ Strongly disagree ☐ Disagree ☐ Somewhat disagree ☐ Neutral ☐ Somewhat agree ☐ Agree ☐ Strongly agree

6. In what other way(s) were CCAs **helpful**?

____

____

____

III. Feasibility

1. I had enough **time** to complete the CCAs activity before my appointment.

☐ Strongly disagree ☐ Disagree ☐ Somewhat disagree ☐ Neutral ☐ Somewhat agree ☐ Agree ☐ Strongly agree

IV. Usability

1. I would **use** CCAs again in a future appointment.

☐ Strongly disagree ☐ Disagree ☐ Somewhat disagree ☐ Neutral ☐ Somewhat agree ☐ Agree ☐ Strongly agree

2. I would **recommend** other teens to use CCAs as well.

☐ Strongly disagree ☐ Disagree ☐ Somewhat disagree ☐ Neutral ☐ Somewhat agree ☐ Agree ☐ Strongly agree

3. Do you have any feedback on how CCAs are **used**?

____

____

____

INTERVIEW GUIDE

[Introductions and interview/ethical guidelines and considerations]

Patient as a participant

1. When you met with your doctor, you set a lifestyle goal to work on over these past 3 weeks.
    a. Do you remember what your goal was?
    b. Did you achieve this goal?
        i. On a scale of 0 to 9, to what degree did you achieve this goal (0 = did not achieve this goal at all; 9 = entirely achieved this goal)?
        ii. On a scale of 0 to 9, how much effort did you make to achieve your goal (0 = did not make any effort; 9 = made a lot of effort)?

2. About your experience in working towards your goal, did you:
    a. Experience anything that made it difficult to achieve your goal?
    b. Experience anything that made it easier to achieve your goal?
    c. Have recommendations for anything that would make it easier to achieve your goal?

3. In the future:
    a. Do you plan to continue (or re-try) working on the goal you made? If no, perhaps work on another lifestyle area/goal? Query for details.
    b. Do you plan to share this goal with your doctor the next time you meet? Why or why not? What about the goal do you plan to discuss?
        i. *Probe:* How did/can other lifestyle habits fit in with the goal that you set?

Patient as a partner

4. *(Only for experimental group)* As a reminder, after your appointment, you completed a survey about goal-setting with your doctor (this was about listening and learning from each other, sharing ideas, agreeing on a goal, and achieving your goal). We asked you to complete this survey because we want to test whether teens who used the *Conversation Cards for Adolescents* had different experiences and conversations with their doctors compared to teens who did not use the cards.

    a. Can you tell me if using the cards affected your medical appointment?
        i. *Probe:* If you go back to when you used the cards in your appointment, did they help you or change things for you in any way (*e.g.,* independency, interaction, involvement, motivation, rapport with your doctor)?
    b. Which of these do you think are most important? Why?

5. Now, I would like to ask you some questions about the way we designed our study.
    a. Do you think working on one goal only was realistic? Why or why not?
    b. Do you think the 3-week time period to achieve your goal was realistic? Why or why not?
    c. What do you think about including parents in this study? Should they have been included more or less? Please explain.
    d. Is there anything about the study that you would change?
        i. *Probe:* Completing the surveys on an iPad *vs* hard-copy, completing the tool activity alone in a separate room, choosing to keep the CC chart note, gift cards amount and source, randomized *vs* non-randomized design?

6. Those are all of the questions I wanted to ask you. Was there anything you wanted to talk with me about that we haven't discussed already?

Thank you so much again for being a part of this study.

Your input was really helpful and appreciated!
